# Psychometric properties of the PROMIS Physical Function item bank in patients receiving physical therapy

**DOI:** 10.1371/journal.pone.0192187

**Published:** 2018-02-12

**Authors:** Martine H. P. Crins, Philip J. van der Wees, Thomas Klausch, Simone A. van Dulmen, Leo D. Roorda, Caroline B. Terwee

**Affiliations:** 1 Amsterdam Rehabilitation Research Center | Reade, Amsterdam, The Netherlands; 2 IQ Healthcare, Radboud University Medical Center, Nijmegen, The Netherlands; 3 Department of Epidemiology and Biostatistics, Amsterdam Public Health Research Institute, VU University Medical Center, Amsterdam, The Netherlands; TNO, NETHERLANDS

## Abstract

**Objectives:**

The Patient-Reported Outcomes Measurement Information System (PROMIS) is a universally applicable set of instruments, including item banks, short forms and computer adaptive tests (CATs), measuring patient-reported health across different patient populations. PROMIS CATs are highly efficient and the use in practice is considered feasible with little administration time, offering standardized and routine patient monitoring. Before an item bank can be used as CAT, the psychometric properties of the item bank have to be examined. Therefore, the objective was to assess the psychometric properties of the Dutch-Flemish PROMIS Physical Function item bank (DF-PROMIS-PF) in Dutch patients receiving physical therapy.

**Design:**

Cross-sectional study.

**Setting and participants:**

805 patients >18 years, who received any kind of physical therapy in primary care in the past year, completed the full DF-PROMIS-PF (121 items).

**Methods:**

Unidimensionality was examined by Confirmatory Factor Analysis and local dependence and monotonicity were evaluated. A Graded Response Model was fitted. Construct validity was examined with correlations between DF-PROMIS-PF T-scores and scores on two legacy instruments (SF-36 Health Survey Physical Functioning scale [SF36-PF10] and the Health Assessment Questionnaire Disability-Index [HAQ-DI]). Reliability (standard errors of theta) was assessed.

**Results:**

The results for unidimensionality were mixed (scaled CFI = 0.924, TLI = 0.923, RMSEA = 0.045, 1th factor explained 61.5% of variance). Some local dependence was found (8.2% of item pairs). The item bank showed a broad coverage of the physical function construct (threshold-parameters range: -4.28–2.33) and good construct validity (correlation with SF36-PF10 = 0.84 and HAQ-DI = -0.85). Furthermore, the DF-PROMIS-PF showed greater reliability over a broader score-range than the SF36-PF10 and HAQ-DI.

**Conclusions:**

The psychometric properties of the DF-PROMIS-PF item bank are sufficient. The DF-PROMIS-PF can now be used as short forms or CAT to measure the level of physical function of physiotherapy patients.

## Introduction

Patient Reported Outcome Measures (PROMs) have become standard instruments to measure patients’ perceived health, and are used to assist in patient-physician shared-decision making and to monitor patients’ health over time. However, its application in daily clinical practice is not without problems. Many traditional PROMs are too long for use in daily clinical practice, and sometimes contain irrelevant and poorly formulated questions [1,2]. The measurement error is often too large to make decisions based on the individual PROM scores and to evaluate changes in individual patients. Furthermore, the scores of traditional PROMs are often difficult to interpret and cannot be compared between the many different existing PROMs [3].

The Patient-Reported Outcomes Measurement Information System (PROMIS^®^) has the potential to overcome some of the shortcomings of existing PROMs [4]. PROMIS is an innovative universally, that is a generic or non-disease specific, applicable set of instruments designed to measure patient-reported health across different populations, including patient populations, in a more efficient way [5,6]. PROMIS instruments are developed using Item Response Theory (IRT) and consist of item banks. These item banks (a set of items [questions] measuring one common construct) can be applied as short forms (fixed length subsets of items out of the item bank) or highly efficient computer adaptive tests (CAT). A CAT is a computer-administered measure in which successive items are selected by a computer algorithm based on responses to previous items. Patients only have to respond to a small number (3–7) of highly informative and relevant items. As a consequence, PROMIS tools are, if applied as short form or CAT, relatively short and the administration time is much less compared to traditional PROMs. Moreover, the CAT application results in estimates with a low measurement error [7]. PROMIS tools were carefully developed in close collaboration with patients and experts, ensuring its content validity. All PROMIS scores are standardized and expressed as T-scores with a population mean of 50 and a Standard Deviation (SD) of 10, enabling easy interpretation. In short, PROMIS instruments are easy to interpret, are less burdensome, have less measurement error, and have better content validity than traditional PROMs [7–9].

The PROMIS Physical Function (PROMIS-PF) item bank is one of the PROMIS instruments which is highly relevant for physical therapists and their patients [10]. Physical function refers to the ability to perform activities of daily living and instrumental activities of daily living [10]. Limitations in physical function are a major concern for elderly and patients with musculoskeletal diseases [11,12]. Physical therapy treatment often focuses on improving physical function. Physical function is therefore frequently a core outcome of treatment. The PROMIS-PF item bank has shown to have less measurement error, stronger content validity and other desirable psychometric properties, compared to traditional physical function PROMs such as the SF-36 Health Survey Physical Functioning scale (SF36-PF10) and the Health Assessment Questionnaire-Disability Index (HAQ-DI) [5,8,9,13,14]. The PROMIS-PF item bank was translated into Dutch-Flemish and showed good psychometric properties in Dutch patients with chronic pain and rheumatoid arthritis [15–18]. However, the PROMIS-PF item bank has not yet been validated in patients receiving physical therapy.

In line with the international PROMIS goals to re-do the calibration of item banks and evaluate its psychometric properties in multiple validation studies and in patients with multiple conditions before an item bank can be used as CAT, the aim of current study was to examine the psychometric properties of the V1.2 Dutch-Flemish PROMIS-PF item bank (DF-PROMIS-PF) in Dutch patients receiving physical therapy in primary care. This is the first study re-doing the calibration of the PROMIS-PF in patients receiving physical therapy. The ultimate aim is to obtain a user-friendly, efficient, precise and valid instrument to measure physical function in patients receiving physical therapy in daily clinical practice and research.

## Methods

### Study participants

For this study Dutch patients (18 years or older) receiving physical therapy in primary care in the past year were invited. Patients were eligible if they provided informed consent.

### Procedures

The study was approved by the local institutional review board of the VU University Medical Center. Physical therapy practices across the Netherlands were approached to recruit patients for the study through the personal network of the authors, and advertisement in a Dutch physical therapy journal. Thereafter, the patients were invited by their physical therapist, by e-mail or flyer, to complete an online questionnaire.

### Measures

The questionnaire included items addressing demographic and clinical characteristics. The questionnaire also included all 121 items of the V1.2 DF-PROMIS-PF. The items cover a wide range of activities, from self-care (activities of daily living) to more complex activities that require a combination of skills. The item bank includes items about functioning of the axial regions (neck and back), the upper and lower extremities, and ability to carry out instrumental activities of daily living (i.e. housework, shopping) [10]. There is no time frame set for the items, but current status is inferred. There are three different 5-point Likert response scales: 1) Unable to do/With much difficulty/With some difficulty/With a little difficulty/Without any difficulty, 2) Cannot do/Quite a lot/Somewhat/Very little/Not at all; and, 3) Cannot do because of health/A lot of difficulty/Some difficulty/A little bit of difficulty/No difficulty at all. Higher scores indicate better function.

In addition, two generic legacy instruments were administered: the SF36-PF10 and the HAQ-DI [19,20]. The Dutch version 2 of the SF36-PF10 was used, which consists of 10 items measuring perceived limitations in a variety of physical activities. Scores of the SF36-PF10 are summed and linearly transformed to range between 0 and 100, with higher scores indicating better physical function [20]. The SF36-PF10 has demonstrated good reliability and validity in Canadian patients with musculoskeletal disorders undergoing physical therapy [21], and in Dutch patients with rheumatoid arthritis [22]. The Dutch version of the HAQ-DI was used, which contains 20 items measuring physical disabilities over the past week in eight categories of daily living. The category scores were averaged to produce a total score ranging from 0 to 3, with higher scores indicating more disability [19]. Disability scores were calculated according the alternative scoring rule indicating that its scores depends on the amount of self-reported difficulty while performing activities, and not on the use of aids or help. The HAQ-DI has demonstrated good reliability and validity within Dutch patients with rheumatoid arthritis and osteoarthritis [23,24].

### Statistical analysis

Demographic and clinical characteristics were described by descriptive statistics. Psychometric analyses were conducted in accordance with the PROMIS analysis plan and were similar to the re-doing of the calibration of the DF-PROMIS-PF in Dutch patients with chronic pain [15,25]. In order to apply an instrument as CAT and to obtain valid scores for CATs and short forms it is important that the items fit an IRT model, because the CAT algorithm and scoring system are based on the parameters of the underlying IRT model. Before applying IRT models, it is important to evaluate the core assumptions of the IRT model: (1) unidimensionality, indicating that the items assess one and only one construct, in this study physical functioning, (2) local independence, indicating that the items are only related to the construct being measured and not to other factors, and (3) monotonicity, indicating that the probability of a affirmative response to the item increases with increasing levels of the underlying construct.

To check unidimensionality, a Confirmatory Factor Analysis (CFA) was fitted using the R-package Lavaan (version 0.5–16) [26,27]. All items were hypothesized to load on a single factor. Model fit was evaluated based on the Comparative Fit Index (CFI), Tucker Lewis Index (TLI) and Root Means Square Error of Approximation (RMSEA). Both scaled and unscaled fit indices were calculated. Since the CFI, TLI and RMSEA are Chi-square based the scaled parameters are considered more exact [28,29]. Model fit criteria include CFI >0.95, TLI >0.95 and RMSEA <0.06 [30]. As an additional approach for assessing unidimensionality, we conducted a Principal Component Analysis (PCA). Sufficient evidence for unidimensionality was considered if the first factor accounts for at least 20% of the variability and when the ratio of the variance explained by the first to the second factor is greater than four [25].

Local independence was evaluated by considering the residual correlation matrix [31]. Residual correlations greater than 0.2 were considered as indicators of possible local dependence [25]. Item pairs which were highly correlated (>0.80) were inspected in more detail with regard to item content and distribution of item response categories. In addition, we inspected model modification indices. Modification indices show improvement in Chi-square if residual correlations of an item pair were left free in the model and thus present a useful tool for identifying problematic items.

Monotonicity was evaluated by estimating a nonparametric Mokken scale with the R-package Mokken [25,32–35]. Fit of model was evaluated by calculating the scalability coefficient H. Monotonicity criteria are met if (1) the scalability coefficients for all item pairs are positive, (2) the scalability coefficients for the items in relation to the scale at issue are at least 0.30, and (3) the scalability coefficient H for the scale is at least 0.30. Higher values for H indicate a better scale. A rule of thumb is that a scale is considered to be strong when H is ≥0.50 [32–35].

When the assumptions of unidimensionality, local independence, and monotonicity were met, the fit of the Graded Item Response model (GRM) was examined, indicating if the IRT model fits to the response data. A logistic GRM was used to estimate item slopes, thresholds, and individual theta scores, using IRT PRO [36]. The item slopes represent the discriminative ability of the items. The item thresholds represent the item difficulties, and locate the items along the measured trait. The theta represents the individual’s physical function score. For standardization and international comparison, the theta scores were transformed into T-scores, where a T-score 50 represents the average score of the general US population, with a SD of 10.

Differential Item Functioning (DIF) analyses evaluate if persons from different groups (for example male vs. female), with similar levels of physical function, respond similar to the items which implies validity of comparisons between the groups at issue [25,37,38]. DIF was evaluated by ordinal logistic regression models in the R package Lordif (version 0.3–3), in which a McFadden’s pseudo R2 change of 2% was used as the critical value to flag for possible DIF [25,39–41]. DIF was evaluated for age (median split) and gender (male vs. female). When items were flagged for DIF, the impact of DIF was examined by plotting item characteristic curves (ICCs) and test characteristic curves (TCCs).

Construct validity indicates whether the item bank really measures the intended construct (physical function). Therefore, construct validity of the DF-PROMIS-PF was evaluated by calculating Spearman correlations between the T-scores of the DF-PROMIS-PF and scores on the two legacy instruments (SF36-PF10 and HAQ-DI). If an instrument measures the intended construct its scores should be highly correlated to scores of other PROMs measuring the same construct. We hypothesized that the DF-PROMIS-PF would have strong correlations with both legacy instruments (r>0.60), but the strongest correlation (r>0.70) with the SF36-PF10, because both DF-PROMIS-PF and SF36-PF10 were both developed for use in a general population whereas the HAQ-DI in the first instance was developed for patients with rheumatoid arthritis.

Reliability indicates whether a measure is precise in estimating the level of the construct, in other words, precise in estimating the physical function T-scores. Reliability within IRT is conceptualized as “information”, in which the fact that measurement precision can differ across levels of the measured trait (θ = Theta) is taken into account [42,43]. Increased information is related to smaller standard errors (SEs) and, therefore, greater measurement precision [42,43]. Plots were overlaid showing SEs, as a parameter of reliability, across the score range of the full DF-PROMIS-PF, the 4-, 6-, 8-, 10-, and 20-item DF-PROMIS-PF Short Forms, and 4-, 6-, 8-, 10-, and 20-item simulated fixed-length CATs. The simulated fixed-length CATs were conducted with use of the R-package catR (version 3.13) [44,45]. Furthermore, the plot included also SEs from the SF36-PF10 and the HAQ-DI, to compare their reliability to the reliability of the DF-PROMIS-PF measures. The distribution of T-scores of the Dutch physiotherapy patients sample was plotted under the reliability plot to show the relation between the reliability of the item bank and the distribution of scores in the sample.

## Results

### Study participants

A total of 805 patients completed the questionnaire. Their demographic and clinical characteristics are summarized in Table 1. The statistical analyses used for checking model assumptions were performed on 753 respondents who had complete DF-PROMIS-PF data because the R packages used cannot handle missing items. However, because IRTPRO can accommodate incomplete data, all 805 patients were used for estimation the IRT model parameters and T-scores were calculated for all 805 patients.

**Table 1:** Demographic and clinical characteristics of the study population.

	Physical therapy patients *(n = 805)*
**Age** *mean (SD) range*	53 (14) 18–88
**Gender** *n (%)*	
Male	331 (41)
Female	474 (59)
**Country of birth** *n (%)*	
Netherlands	761 (95)
Other	44 (5)
**Educational level** *n (%)*	
Less than High School degree	21 (3)
High School degree	82 (10)
Some college	301 (37)
College degree	37 (5)
Advanced degree	364 (45)
**Body region of treatment** *n (%)*	
Head	14 (2)
Breast/abdomen	25 (3)
Neck/upper back	152 (19)
Shoulders/upper arm	113 (14)
Elbow/forearm/hand	23 (3)
Low back	157 (20)
Pelvis/hip/upper leg	76 (9)
Knee	86 (11)
Lower leg/ankle/foot	52 (6)
More than 1 region	107 (13)
**Disorder type for treatment** *n (%)*	
Disorder of muscles, bones or joints without surgery	391 (49)
Recovery after surgery	100 (12)
Condition resulting from an accident without surgery	70 (9)
Cardiac, vascular or lymphatic disorder	25 (3)
Pulmonary affection	20 (2)
Other internal disorder	4 (1)
Neurological disorder	15 (2)
Gynaecological disorder	7 (1)
Disorder with no known cause	11 (1)
Rheumatic disease	17 (2)
Osteoarthritis	45 (6)
Other	100 (12)
**Duration of pain** *n (%)*	
0–3 months	126 (16)
3–6 months	116 (14)
6–12 months	166 (21)
1–2 years	146 (18)
2–5 years	85 (10)
>5 years	166 (21)
**T-score of the PROMIS Physical Function item bank** *mean (SD) range*	48.2 (9.4) 21.4–73.5
**Legacy instruments** *mean (SD)*	
SF36-PF10 *(n = 710)*	75.2 (26)
HAQ-DI *(n = 739)*	0.4 (0.5)

SF36-PF10 = Short Form Health Survey Physical Functioning (range 0–100, higher scores indicate better physical function); HAQ-DI = Health Assessment Questionnaire-Disability Index (range 0–3, higher scores indicate less physical functioning).

### Model assumptions

The results for unidimensionality were mixed: The CFA analyses showed unscaled fit indices of CFI = 0.982, TLI = 0.982 and RMSEA = 0.091, and scaled indices of CFI = 0.924, TLI = 0.923 and RMSEA = 0.045. The scaled CFI and TLI did not met the criterion of >0.95, while the scaled RMSEA did met the criterion of <0.06. Furthermore, the first factor accounted for 61.5% of the variance and the ratio of the variance explained by the first to the second factor was 7.6, well above the criterion of 4. Altogether showing sufficient unidimensionality.

Some violations of local independence were found: The residual correlation matrix showed that 592 out of 7260 item pairs (8.2%) were flagged for local dependence (S1 Appendix). It was found that upper extremity related items addressing easy activities (e.g. tooth brushing) were often highly residual correlated with items related to running. Further inspection of these item pairs showed that only few patients answered ‘unable to do’ or ‘with much difficulty’ to these easy items, while the distribution of responses to the running items were more balanced. This might explain the residual correlation. As a further approach to evaluate local independence, modification indices were examined to see if freeing the residual correlations would improve the model. It was found that the item pairs with the highest modification indices show strong overlap in item content (S1 Appendix). Many items related to running, lifting, and items with very similar wording were residually related, which may indicate a second factor in the data. Therefore, we examined if items related to running or lifting could form a factor by themselves. A CFA on 11 items related to walking or running resulted in scaled CFI = 0.98, TLI = 0.98, and RMSEA = 0.16. A CFA on 12 items related to lifting resulted in scaled CFI = 0.99, TLI = 0.98, and RMSEA = 0.11. The RMSEA values were considered too high to consider a second factor.

No violations of monotonicity were found. The scalability coefficients for all item pairs were positive, only one item had a scalability coefficient <0.30 and the scalability coefficient H for the full scale was 0.57, suggesting strong scalability.

### IRT model parameters

The GRM item slope parameters ranged from 1.36 to 4.29 (mean 2.74) and the item threshold parameters ranged from -4.28 to 2.33, indicating good coverage of the physical function construct. The mean T-score of the DF-PROMIS-PF for the Dutch sample was 48.2 (SD = 9.4), with a range from 21.4 to 73.5, indicating a slightly lower average level of physical function in Dutch patients currently receiving physical therapy treatment or had completed physical therapy treatment in the past year compared to persons from the US general population, with large variation among patients.

### Differential Item Functioning

Only two out of 121 Dutch-Flemish PROMIS Physical Function items were flagged for DIF for age and fourteen for DIF for gender (S2 Appendix). However, investigation of ICCs and TCCs showed that their impact on the T-scores was negligible.

### Construct validity

The DF-PROMIS-PF correlated strongly with both the SF36-PF10 (r = 0.84)) and the HAQ-DI (r = -0.85), as expected. The correlation with the SF-PF10 was, however, not higher than the correlation with the HAQ-DI.

### Reliability

**Fig 1:**
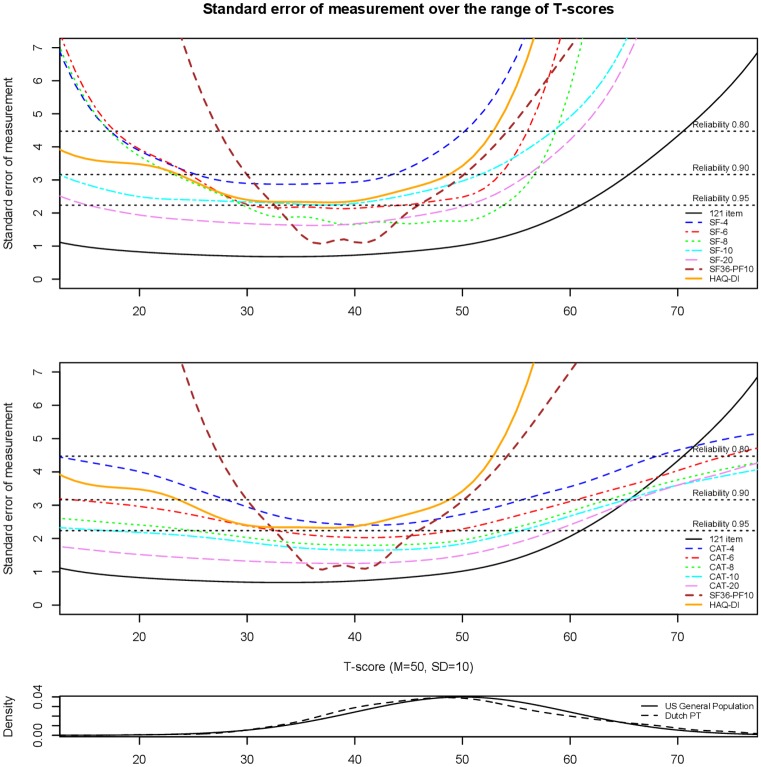
The two upper plots show the standard errors of the total Dutch-Flemish PROMIS Physical Function item bank (121 item), the 4-, 6-, 8-, 10-, and 20-item short forms (SFs) and CATs, and the SF36-PF10 and HAQ-DI, respectively. The horizontal axis represents the different physical function abilities with T = 50 representing the mean of the US general population with a standard deviation of 10. The vertical axis represents the standard error (reliability), with reference reliabilities of 0.80, 0.90 and 0.95. The lower the curve, the greater the reliability. The lower plot shows the distribution of the Dutch physiotherapy patients (Dutch PT) sample and the US general population sample along the T-score scale.

Fig 1 shows plots of the SEs across the range of the full DF-PROMIS-PF, short forms, simulated CATs, and the SF36-PF10 and HAQ-DI. The reliability of the total item bank was greater than 0.95 for the range of the scale where most of the study sample was located (between T-scores 35–60), indicating very good reliability. The plots demonstrate that CATs show greater reliability than the short forms. Furthermore, the DF-PROMIS-PF instruments show greater reliability than the SF36-PF10 and HAQ-DI across most of the trait.

## Discussion

This is the first validation study of PROMIS in a physical therapy population. The results of the current study add to the evidence on the psychometric properties of the DF-PROMIS-PF. The results supported sufficient unidimensionality, showed some local dependence, good monotonicity, good IRT model fit, good coverage of the construct of physical function and negligible DIF for age and gender. Furthermore, good construct validity and high reliability across the physical function construct was found. Although further improvement of the item bank may be possible, we consider the psychometric properties of the DF-PROMIS-PF sufficient to measure the level of physical function of physiotherapy patients if being applied as short forms or CAT.

The average age (53yr) and percentages of females (60%) in our sample matches with the average physical therapy patient population in the Netherlands in 2015, but the percentage of patients with complaints longer than 6 months was much higher in our sample (70% compared to 25% in the Netherlands) [46]. Due to the selection procedure of the study, we do not know how many patients were still under treatment. The mean T-score (48.2) was only slightly lower (0.18SD) than the average US general population, which could indicate that many patients had completed their treatment.

Our results regarding the psychometric properties of the DF-PROMIS-PF were similar to those of previous studies of the PROMIS-PF. Previous studies in different patient populations and in people from the general population also found small problems with the IRT assumptions unidimensionality and local dependence (S3 Appendix) [10,15,17,47,48]. On the contrary, Paz et al. found good results regarding the IRT assumptions in Spanish speaking persons (S3 Appendix) [49]. Regarding local dependence, 8% local dependence was found in the current study, whereas this was 6% in Dutch chronic pain patients and 10% in Spanish speaking persons [15,48]. The current study showed that sparsity in the item response categories (few patients who answered ‘unable to do’ or ‘with much difficulty’ to the easy items) was a major cause of misfit and local dependence. Sparsity in item response categories was also found by Rose et al. and Hung et al. [10,47]. This indicates that some of the ‘easy’ items such as ‘are you able to brush your teeth’ may be less relevant for these populations. Furthermore, in the current study there were items that showed high overlap in item content, mostly regarding running and lifting. However, analyses showed that there was no running or lifting factor present besides the physical function factor.

In the current study items with DIF with respect to age and gender were found, however their impact on the physical function scores was negligible. Previous studies on the PROMIS-PF also showed no or minimal impact of DIF for age and gender [10,15,17]. Overall, we conclude that the DF-PROMIS-PF can be used across physical therapy patients that differ in age or gender.

The current study supports the construct validity of the DF-PROMIS-PF, by showing strong correlations between the DF-PROMIS-PF and the traditionally used SF36-PF10 (r = 0.84) and HAQ-DI (r = -0.85). This was also found by Oude Voshaar et al., who found similar strong correlations between the DF-PROMIS-PF and both the SF36-PF10 (r = 0.84) and the HAQ-DI (r = -0.76) [16].

The current study as well as several previous studies showed that the PROMIS-PF measures have good reliability; the reliability of the total item bank as well as the short forms and CATs were greater than 0.80 or even 0.90 or 0.95 for the range of the scale where the study samples were located [10,15,16]. These studies also showed that CATs outperform short forms [10,15,16]. Furthermore, the current and previous studies showed that the PROMIS-PF measures have better precision (smaller measurement error) over a broader score-range compared to the SF36-PF10 and the HAQ-DI [8,9,14]. Moreover, PROMIS CATs are more efficient and more feasible for use in daily clinical practice than the SF36-PF10 and HAQ-DI. The administration time for the physician, which is now a big problem within physical therapy, is much less with the PROMIS CATs compared to other PROMs. A recent study in patients undergoing meniscal surgery showed that the majority (89%) of the patients completed the PROMIS-PF CAT after answering 4 items [50]. Moreover, PROMIS T-scores are easy to interpret and can easily be used for patient-physician communication and goalsetting, for monitoring patients and for monitoring the progress of the treatment on physical function level.

Were the current study focused on examining the psychometric properties of the whole item bank and showed sufficient properties of the DF-PROMIS-PF to be used as CAT, future research is recommended on known-groups validity, test-retest reliability and responsiveness of the CAT, because the CAT would most likely be used in pre- and post-intervention measurements. A recent study in orthopedic reconstructions patients showed that the PROMIS-PF CAT outperformed the legacy instruments Knee injury and Osteoarthritis Outcome Score Joint Replacement (KOOS-JR) and Hip disability and Osteoarthritis Outcome Score Joint Replacement (HOOS-JR) with respect to responsiveness [51].

The psychometric properties of the DF-PROMIS-PF item bank are sufficient for use as short forms or CAT to measure the level of physical function in Dutch physical therapy practice. Using the highly efficient DF-PROMIS-PF CAT in clinical practice is considered feasible with little administration time, and has the potential for standardized and routine patient monitoring across a wide range of patients receiving physical therapy.

## Supporting information

S1 Appendix Results of checking IRT model assumptions. (DOCX)

S2 Appendix Differential Item Functioning (DIF) results regarding age and gender. (DOCX)

S3 Appendix Overview of results regarding the IRT assumptions of the PROMIS-PF item bank of the current and previous studies. (DOCX)
