# Supplementary material for: Psychometric properties of the PROMIS Physical Function item bank in patients receiving physical therapy
Source: PLoS One. 2018 Feb 12;13(2):e0192187. doi: 10.1371/journal.pone.0192187 (PMC5809015; doi:10.1371/journal.pone.0192187)
Supplement: S1 Appendix — (DOCX) [file pone.0192187.s001.docx]

**S1 Appendix. Results of checking IRT model assumptions**

The ten item pairs with the highest local dependence, sorted by the highest residual correlations.

| **Item 1 code** | **Item description** | **Item 2 code** | **Item description** | **Residual correlation** | **Polychorical correlation** | **Scaled modification indices** |
| --- | --- | --- | --- | --- | --- | --- |
| PFA39r1 | Are you able to run at a fast pace for two miles (3 km)? | PFC49 | Are you able to water a house plant? | -0.575 | 0.048 | 8.416 |
| PFA50 | Are you able to brush your teeth? | PFC7r1 | Are you able to run five miles (8 km)? | -0.562 | 0.129 | 6.916 |
| PFB29 | Are you able to lift a full cup or glass to your mouth? | PFC7r1 | Are you able to run five miles (8 km)? | -0.545 | 0.108 | 4.741 |
| PFA39r1 | Are you able to run at a fast pace for two miles (3 km)? | PFB29 | Are you able to lift a full cup or glass to your mouth? | -0.537 | 0.091 | 5.246 |
| PFB37 | Are you able to turn faucets on and off? | PFC7r1 | Are you able to run five miles (8 km)? | -0.510 | 0.164 | 6.919 |
| PFA39r1 | Are you able to run at a fast pace for two miles (3 km)? | PFA50 | Are you able to brush your teeth? | -0.507 | 0.157 | 7.298 |
| PFC49 | Are you able to water a house plant? | PFC7r1 | Are you able to run five miles (8 km)? | -0.499 | 0.149 | 5.938 |
| PFA19r1 | Are you able to run or jog for two miles (3 km)? | PFB29 | Are you able to lift a full cup or glass to your mouth? | -0.498 | 0.158 | 4.923 |
| PFB16 | Are you able to press with your index finger (for example ringing a doorbell)? | PFC7r1 | Are you able to run five miles (8 km)? | -0.492 | 0.225 | 2.328 |
| PFA39r1 | Are you able to run at a fast pace for two miles (3 km)? | PFB16 | Are you able to press with your index finger (for example ringing a doorbell)? | -0.472 | 0.218 | 3.004 |

The ten items with most sparsity in the item response categories, sorted by the highest skewness values.

| **Item code** | **Item** | **Unable to do** | **With much difficulty** | **With some difficulty** | **With a little difficulty** | **Without any difficulty** | **Skewness** |
| --- | --- | --- | --- | --- | --- | --- | --- |
| PFB16 | Are you able to press with your index finger (for example ringing a doorbell)? | 0 | 3 | 6 | 7 | 737 | -8.273 |
| PFA50 | Are you able to brush your teeth? | 0 | 1 | 4 | 16 | 732 | -7.655 |
| PFC49 | Are you able to water a house plant? | 0 | 2 | 5 | 16 | 730 | -7.417 |
| PFB18 | Are you able to shave your face or apply makeup? | 4 | 1 | 9 | 20 | 719 | -6.827 |
| PFB29 | Are you able to lift a full cup or glass to your mouth? | 0 | 0 | 8 | 13 | 732 | -6.786 |
| PFB20 | Are you able to cut a piece of paper with scissors? | 0 | 3 | 6 | 19 | 725 | -6.726 |
| PFB19 | Are you able to squeeze a new tube of toothpaste? | 0 | 3 | 11 | 17 | 722 | -5.944 |
| PFB37 | Are you able to turn faucets on and off? | 0 | 5 | 8 | 25 | 715 | -5.724 |
| PFB21 | Are you able to pick up coins from a table top? | 2 | 3 | 8 | 36 | 704 | -5.697 |
| PFB31 | Are you able to open car doors? | 0 | 2 | 9 | 25 | 717 | -5.653 |

Cat 1 to Cat 5 = item response categories 1 to 5

The ten item pairs with the highest modification indices.

| **Item 1 code** | **Item 1** | **Item 2 code** | **Item 2** | **Residual correlation** | **Polychorical correlation** | **Scaled modification indices** |
| --- | --- | --- | --- | --- | --- | --- |
| PFC33r1 | Are you able to run ten miles (16 km)? | PFC7r1 | Are you able to run five miles (8 km)? | 0.243 | 0.925 | 210.178 |
| PFA19r1 | Are you able to run or jog for two miles (3 km)? | PFA39r1 | Are you able to run at a fast pace for two miles (3 km)? | 0.224 | 0.908 | 200.999 |
| PFB39r1 | Are you able to reach and get down a 5 pound (2 kg) object from above your head? | PFB56r1 | Are you able to lift one pound (0.5 kg) to shoulder level without bending your elbow? | 0.259 | 0.915 | 146.803 |
| PFB24 | Are you able to run a short distance, such as to catch a bus? | PFC13r1 | Are you able to run 100 yards (100 m)? | 0.182 | 0.921 | 128.994 |
| PFA39r1 | Are you able to run at a fast pace for two miles (3 km)? | PFC7r1 | Are you able to run five miles (8 km)? | 0.215 | 0.897 | 121.099 |
| PFB14 | Are you able to take a tub bath? | PFC54 | Does your health now limit you in getting in and out of the bathtub? | 0.230 | 0.930 | 117.454 |
| PFC12 | Does your health now limit you in doing two hours of physical labor? | PFC35 | Does your health now limit you in doing eight hours of physical labor? | 0.157 | 0.921 | 109.688 |
| PFB28r1 | Are you able to lift 10 pounds (5 kg) above your shoulder? | PFB39r1 | Are you able to reach and get down a 5 pound (2 kg) object from above your head? | 0.231 | 0.907 | 97.784 |
| PFA1 | Does your health now limit you in doing vigorous activities, such as running, lifting heavy objects, participating in strenuous sports? | PFB7 | Does your health now limit you in doing strenuous activities such as backpacking, skiing, playing tennis, bicycling or jogging? | 0.156 | 0.902 | 95.551 |
| PFB32 | Are you able to stand unsupported for 10 minutes? | PFB42 | Are you able to stand unsupported for 30 minutes? | 0.220 | 0.925 | 88.275 |
