# Supplementary material for: Psychometric properties of the PROMIS Physical Function item bank in patients receiving physical therapy
Source: PLoS One. 2018 Feb 12;13(2):e0192187. doi: 10.1371/journal.pone.0192187 (PMC5809015; doi:10.1371/journal.pone.0192187)
Supplement: S2 Appendix — (DOCX) [file pone.0192187.s002.docx]

**S2 Appendix. Differential Item Functioning (DIF) results regarding age and gender**

| **DIF for:** | **Item code** | **Item** |
| --- | --- | --- |
| **Age** | PFB29 | Are you able to lift a full cup or glass to your mouth? |
|  | PFC33r1 | Are you able to run ten miles (16 km)? |
| **Gender** | PFA12 | Are you able to push open a heavy door? |
|  | PFA14r1 | Are you able to carry a heavy object (over 10 pounds /5 kg)? |
|  | PFA17 | Are you able to reach into a high cupboard? |
|  | PFA18 | Are you able to use a hammer to pound a nail? |
|  | PFA22 | Are you able to open previously opened jars? |
|  | PFA28 | Are you able to open a can with a hand can opener? |
|  | PFA29r1 | Are you able to pull heavy objects (10 pounds/ 5 kg) towards yourself? |
|  | PFA47 | Are you able to pull on trousers? |
|  | PFA52 | Are you able to tie your shoelaces |
|  | PFB17 | Are you able to put on and take off your socks? |
|  | PFB28r1 | Are you able to lift 10 pounds (5 kg) above your shoulder? |
|  | PFB30 | Are you able to open a new milk carton? |
|  | PFB39r1 | Are you able to reach and get down a 5 pound (2 kg) object from above your head? |
|  | PFA16r1 | Are you able to dress yourself, including tying shoelaces and buttoning your clothes? |
