# Supplementary material for: Psychometric properties of the PROMIS Physical Function item bank in patients receiving physical therapy
Source: PLoS One. 2018 Feb 12;13(2):e0192187. doi: 10.1371/journal.pone.0192187 (PMC5809015; doi:10.1371/journal.pone.0192187)
Supplement: S3 Appendix — (DOCX) [file pone.0192187.s003.docx]

**S3 Appendix. Overview of results regarding the IRT assumptions of the PROMIS-PF item bank of the current and previous studies**

|  | | **Unidemensionality** | | | | | | **Local Dependence** | **Monotonicity** |
| --- | --- | --- | --- | --- | --- | --- | --- | --- | --- |
|  |  | **Unscaled indices** | | | **Scaled indices** | | |  |  |
| **Study** | **Population** | **CFI** | **TLI** | **RMSEA** | **CFI** | **TLI** | **RMSEA** | **%** | **H** |
| Current study | Dutch physical therapy patients | 0.982 | 0.982 | 0.091 | 0.924 | 0.923 | 0.043 | 8 | 0.57 |
| Crins et al. 2017 | Dutch chronic pain patients | 0.976 | 0.976 | 0.122 | * | * | * | 6 | 0.56 |
| Paz et al. 2013 | Spanish speaking general population | * | * | * | 0.971 | 0.970 | 0.052 | 10 | * |

* Not applicable
